# Long‐Term Quality of Life Trend after Subthalamic Stimulation for Parkinson's Disease: An Updated Systematic Review and Meta‐Analysis

**DOI:** 10.1002/mdc3.70025

**Published:** 2025-03-05

**Authors:** Luis Otávio Nogueira, Angela Maria Sandini Corso, Louise Dalla Corte Dallé, Vanio L.J. Antunes, Matheus de M. Fernandes, Isabella S.M. Rabelo, Marcus V. Della Coletta, Carolina Candeias da Silva, Lorena Broseghini Barcelos, Henrique Ballalai Ferraz, Robson Luis Oliveira de Amorim, Dayany Leonel Boone

**Affiliations:** ^1^ Amazonas State University Medicine Department Manaus Brazil; ^2^ Paraná Federal University Curitiba Brazil; ^3^ Federal University of Health Sciences of Porto Alegre Medicine Department Porto Alegre Brazil; ^4^ State University of Rio Grande do Norte Mossoró Brazil; ^5^ State University of Ceará Fortaleza Brazil; ^6^ Federal University of São Paulo Department of Neurology and Neurosurgery São Paulo Brazil; ^7^ Health Sciences Faculty Federal University of Amazonas Manaus Brazil

**Keywords:** STN‐DBS, Parkinson's disease, meta‐analysis, quality of life, PDQ

## Abstract

**Background:**

Deep brain stimulation of the subthalamic nucleus (STN‐DBS) is a well‐established treatment for Parkinson's Disease (PD). However, the long‐term trajectory of Quality of Life (QoL) following STN‐DBS remains underexplored.

**Objectives:**

We aimed to conduct a systematic review and meta‐analysis to assess QoL trends up to five years after STN‐DBS.

**Methods:**

We systematically searched PubMed, Embase, and Cochrane databases from inception to August 2024 for studies involving PD patients treated with bilateral STN‐DBS, evaluating QoL using the Parkinson's Disease Questionnaire (PDQ), with a minimum follow‐up of 12 months post‐surgery. Continuous outcomes were pooled using standardized mean differences (SMD), and statistical analyses were conducted using R version 4.3.2.

**Results:**

Out of 4106 screened articles, 42 studies with a total of 2767 patients were included in the meta‐analysis. QoL improvements were observed up to 36 months post‐surgery (SMD 0.83; 95% CI 0.29 to 1.37), followed by a decline to pre‐operative levels at 60 months (SMD ‐0.06; 95% CI ‐0.26 to 0.15). Subdomain analysis at 60 months revealed significant deterioration in cognitive function and communication. Meta‐regression indicated that QoL improvements were independent of clinical and sociodemographic factors such as age, sex, and disease duration; however, there was a correlation with mean baseline PDQ (*P* = 0.01).

**Conclusions:**

This meta‐analysis provides long‐term QoL trends following STN‐DBS, highlighting a further need to explore the factors driving the decline in QoL and develop strategies to mitigate this deterioration.

Parkinson's Disease (PD) is a progressive disorder characterized by neuronal degeneration and intraneuronal misfolded α‐synuclein in specific central and peripheral nervous system regions.^1^ This process leads to both motor and non‐motor symptoms (NMS),^2^ which collectively contribute to a decreased quality of life (QoL).^2^ Deep brain stimulation of the subthalamic nucleus (STN‐DBS) is a well‐established and efficacious treatment for disabling PD motor complications that are not well‐controlled with the best available medical therapy.^3^ Studies on the long‐term effects of STN‐DBS have mainly focused on motor symptoms, documenting sustained benefits on bradykinesia, rigidity and tremor for up to 10 years.^4^ However, these motor improvements do not necessarily translate into an improvement in QoL, which is influenced by psychosocial factors and the persistence of specific NMS.^5^ Some patients report dissatisfaction after surgery despite improvement in motor functions.^6^

Considering the invasive procedure of deep brain stimulation, identifying the time course of QoL after surgery is essential and may help develop strategies to prevent or delay its impairment on a long‐term follow‐up, since PD has a progressive nature. A previous meta‐analysis^7^ of patients undergoing STN‐DBS reported a significant QoL improvement for up to three years after surgery, with subsequent decrements in these gains at a five‐year follow‐up when QoL returned to preoperative status. However, as mentioned by the authors,^7^ their analysis did not include enough patients to come to a confident conclusion, since only three studies reported QoL measures at 36 months (84 patients) and at 60 months (76 patients), which means QoL trend after STN‐DBS, specially three years and beyond, remains unknown. Recently, other larger cohorts have been published,^8^, ^9^, ^10^, ^11^ which may strengthen the power of pooled outcomes.

Furthermore, a recent study found that QoL outcomes were stable in patients that received STN‐DBS at a 5‐year follow‐up, while worsened in patients treated with standard‐of‐care medication.^11^ Their result was mainly driven by the mobility domain of Parkinson's Disease Questionnaire (PDQ)‐8. To our knowledge, no meta‐analysis has systematically evaluated each of the eight domains of the PDQ after DBS surgery, which is important to identify the necessary future interventions to maintain QoL improvement over the years. Therefore, we aimed to perform an updated systematic review and meta‐analysis to identify accurately QoL trend up to five years after bilateral STN‐DBS and analyze its components, as well as the influence of clinical and sociodemographic factors on QoL results.

## Methods

This meta‐analysis was registered in PROSPERO with code CRD42024581755. This study was performed in accordance with the Preferred Reporting Items for Systematic Reviews and Meta‐Analysis (PRISMA) Statement and the recommendations of the Cochrane Collaboration Handbook for Systematic Reviews of Interventions.^12^, ^13^

### Eligibility Criteria

Inclusion in this meta‐analysis was restricted to studies that met all the following eligibility criteria: (1) randomized trials or nonrandomized cohorts; (2) enrolling adult patients diagnosed with PD that received bilateral subthalamic stimulation; (3) evaluating QoL with Parkinson's Disease Questionnaire (PDQ)‐39 or its shorter version, the PDQ‐8; (4) providing all PDQ means and standard deviations values; (5) with a follow‐up period of at least 12 months after surgery. Studies were then categorized for the follow‐up analysis into four groups: 12, 24, 36 or 60 months of follow‐up. We excluded studies that: (1) had overlapping patient populations during identical follow‐up period; (2) were not written in English; (3) fulfilled the inclusion criteria but did not fit into one of the groups of follow‐ups.

### Search Strategy

We performed an electronic search including PubMed, Embase and Cochrane Central Register of Controlled Trials from inception to August 2024. We used the following search strategy: (“deep brain stimulation” OR “Subthalamic stimulation” OR “STN‐DBS”) AND (“Quality of Life” OR “Parkinson Disease Questionnaire” OR “PDQ‐39” OR “PDQ8”) AND (“Parkinson”). Two investigators (L.O.N. and A.M.S.C.) screened articles independently, first by title and abstract, then by full text, to determine eligibility for final inclusion. At each stage of screening any differences between reviewers were discussed, and a consensus decision for eligibility and inclusion was made for all articles.

### Main Endpoint

We aimed to assess the long‐term QoL trend after bilateral STN‐DBS. The 39‐item PDQ is the most thoroughly tested and applied questionnaire to assess QoL in PD.^14^ A summary index (SI) ranging 0–100 can be calculated to represent the global health‐related (HR)QoL, with higher scores representing worse QoL.^15^ This index has been used to evaluate the overall effect of different treatments on functioning and well‐being and has shown high levels of reliability and validity across different cultural settings.^16^ A shorter version of the PDQ‐39 is the PDQ‐8, which consists of eight items, each of which represents a subscale of the PDQ‐39. The PDQ‐8 provides lower reliability and validity than the PDQ‐39; however, it is quicker to administer.^17^

### Quality Assessment

The risk of bias in each study was assessed independently by two authors (A.M.S.C. and I.S.M.R.) using Cochrane's tools for assessing risk of bias in randomized trials (RoB 2)^18^ and in non‐randomized studies (ROBINS‐I).^19^ Disagreements were resolved by arbitration by a third author (L.O.N.). The risk of bias plot was created with the Risk of Bias Visualization (ROBVIS) tool.^20^ A funnel plot to assess publication bias was generated using R and analyzed by visual inspection and Egger's test, in accordance with Cochrane handbook.

### Meta‐Analysis

Two authors (L.O.N. and M.M.F.) extracted outcome data independently, and disagreements were resolved by consensus. All patients were analyzed according to per protocol principle. In total, we conducted 12 meta‐analyses. Four of them for each follow‐up period (12, 24, 36 and 60 months) and eight more meta‐analyses were conducted for each subscale of the PDQ to determine how each domain improves or worsens 5 years after STN‐DBS. To avoid possible heterogeneity because of the two different PDQ versions, continuous endpoints were pooled using the standardized mean difference (SMD) with 95% confidence intervals (CI) with Inverse Variance (IV) method. *P* values <0.05 were considered statistically significant. Cochran's Q test and I^2^ statistics were used to assess heterogeneity. Values of *P* < 0.10 and I^2^ > 25% were considered significant for heterogeneity.

To enhance the robustness of our findings, we performed a subgroup analysis of all follow‐up periods, including only studies that used the PDQ‐39 to assess QoL, with the results pooled using mean difference (MD). Additionally, we conducted a meta‐regression to explore the impact of the mean baseline Unified Parkinson's Disease Rating Scale (UPDRS) Part II, Part III (ON state), Part III (OFF state), Part IV, OFF‐to‐ON MDS‐UPDRS Part III change, levodopa equivalent daily dose (LEDD), mean baseline PDQ score, male percentage, mean age and mean disease duration at surgery on QoL outcomes at 12 and 24 months following STN‐DB, when at least 10 studies provided the necessary data.^13^ The Restricted Maximum Likelihood (REML) random‐effects models were used for all outcomes, and R version 4.3.2 was used for statistical analyses.

## Results

### Study Selection and Characteristics

**Figure 1:**
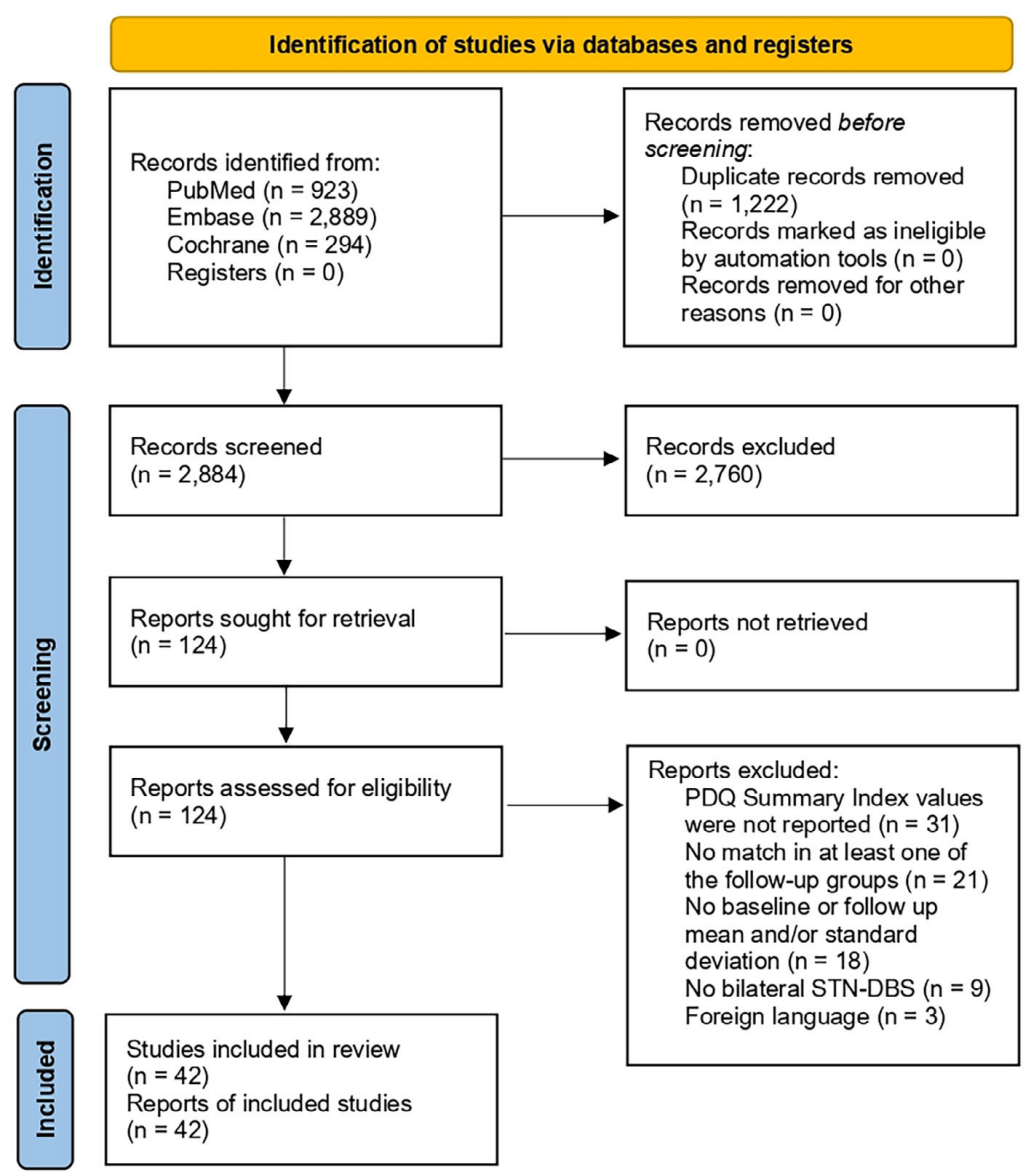
PRISMA flow diagram of study screening and selection.

We identified 4106 reports in the initial database search (Fig. 1). Of these, 124 were fully screened according to the inclusion criteria, and a total of 42 studies and 2767 patients were included in the final analysis, comprising six randomized controlled trials. These studies were conducted between 2005, when QoL began to be used as an outcome variable in DBS studies, and 2024. PDQ‐8 was used for QoL assessment in only four of them.^11^, ^21^, ^22^, ^23^ Other characteristics of the included studies are shown in Table S1.

### Changes in QoL at Different Time Points: Meta‐Analysis

#### 12 Months

The effect of deep brain stimulation (DBS) on the QoL in patients with Parkinson's disease was evaluated over a 12‐month follow‐up period in 34 studies, with a sample of 1992 patients (Fig. S1). The mean age across studies was 60.4 years, and the mean disease duration was 11.0 years at surgery. Four studies did not report the mean disease duration at the time of surgery. QoL scores significantly improved in patients post‐DBS compared to pre‐DBS (SMD 1.26; 95% CI 0.88 to 1.63; I^2^ = 94%).

#### 24 Months

**Figure 2:**
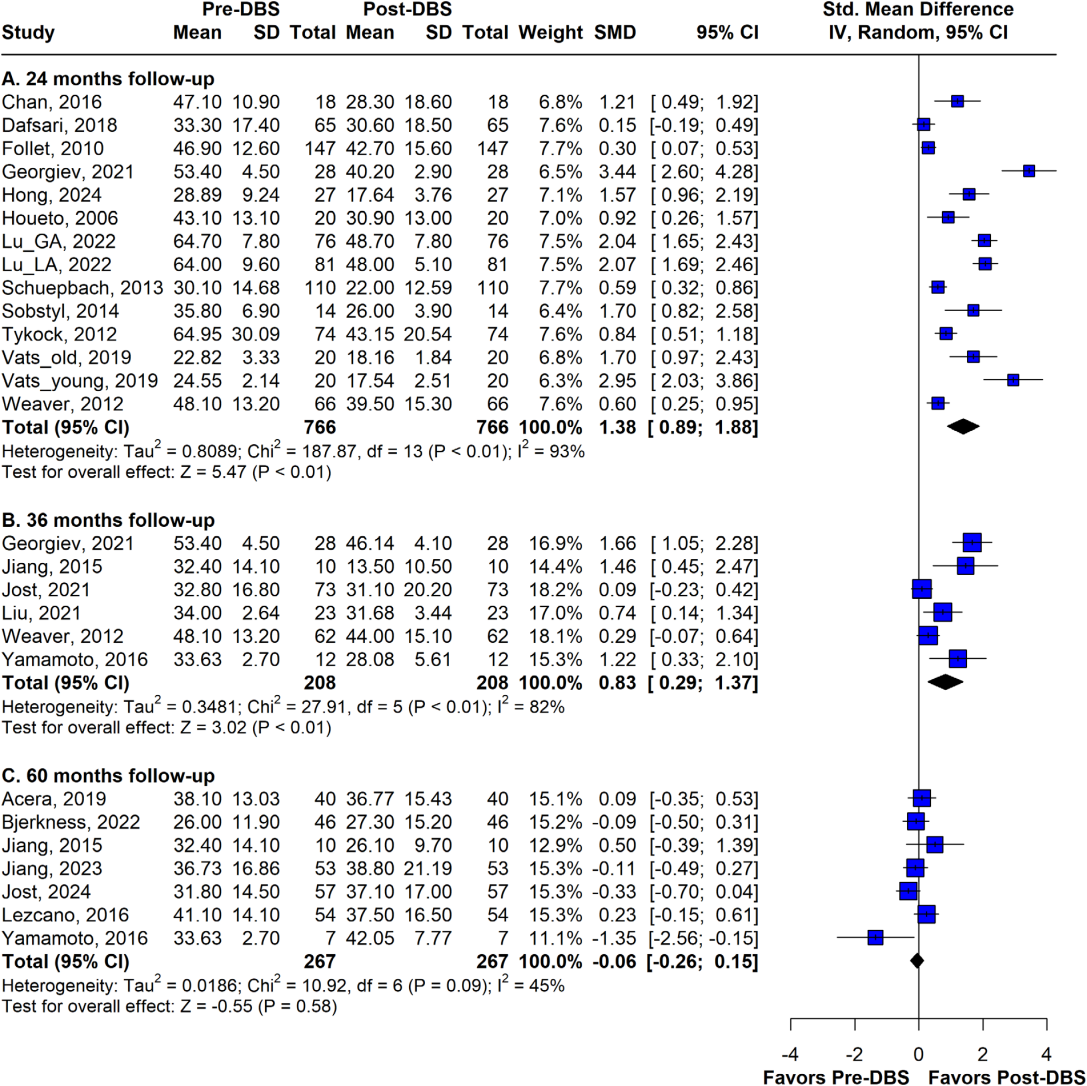
Forest plot of the follow‐up period of 24, 36 and 60 months showing the SMD and the weights for the random effects analysis of each study. DBS, deep brain stimulation; SMD, standardized mean difference.

The 24‐month follow‐up data from 12 studies (*n* = 766) demonstrated a significant improvement in QoL following DBS compared to baseline scores (SMD 1.38; 95% CI 0.89 to 1.88; I^2^ = 93%), as shown in Figure 2. The analysis revealed that, on average, patients were 59.4 years old at the time of surgery, with a disease duration of approximately 10.2 years. Two studies did not report the mean disease duration of their sample.

#### 36 Months

In a 36‐month follow‐up analysis of six studies involving 208 patients, the mean age at the time of surgery was 62.4 years, with an average disease duration of 10.6 years. This meta‐analysis still reveals a significant result (SMD 0.83; 95% CI 0.29 to 1.37; I^2^ = 82%), as illustrated in Figure 2.

#### 60 Months

For the 60‐month follow‐up, seven studies (n = 267) were included in the analysis (Fig. 2). The mean age across these studies was 61.6 years, with a mean disease duration of 11.6 years at the time of surgery. Over this period, the QoL declined to a level not significantly different from baseline (SMD −0.06; 95% CI −0.26 to 0.15; I^2^ = 45%).

### PDQ Domains after 60 Months: Meta‐Analyses

**Figure 3:**
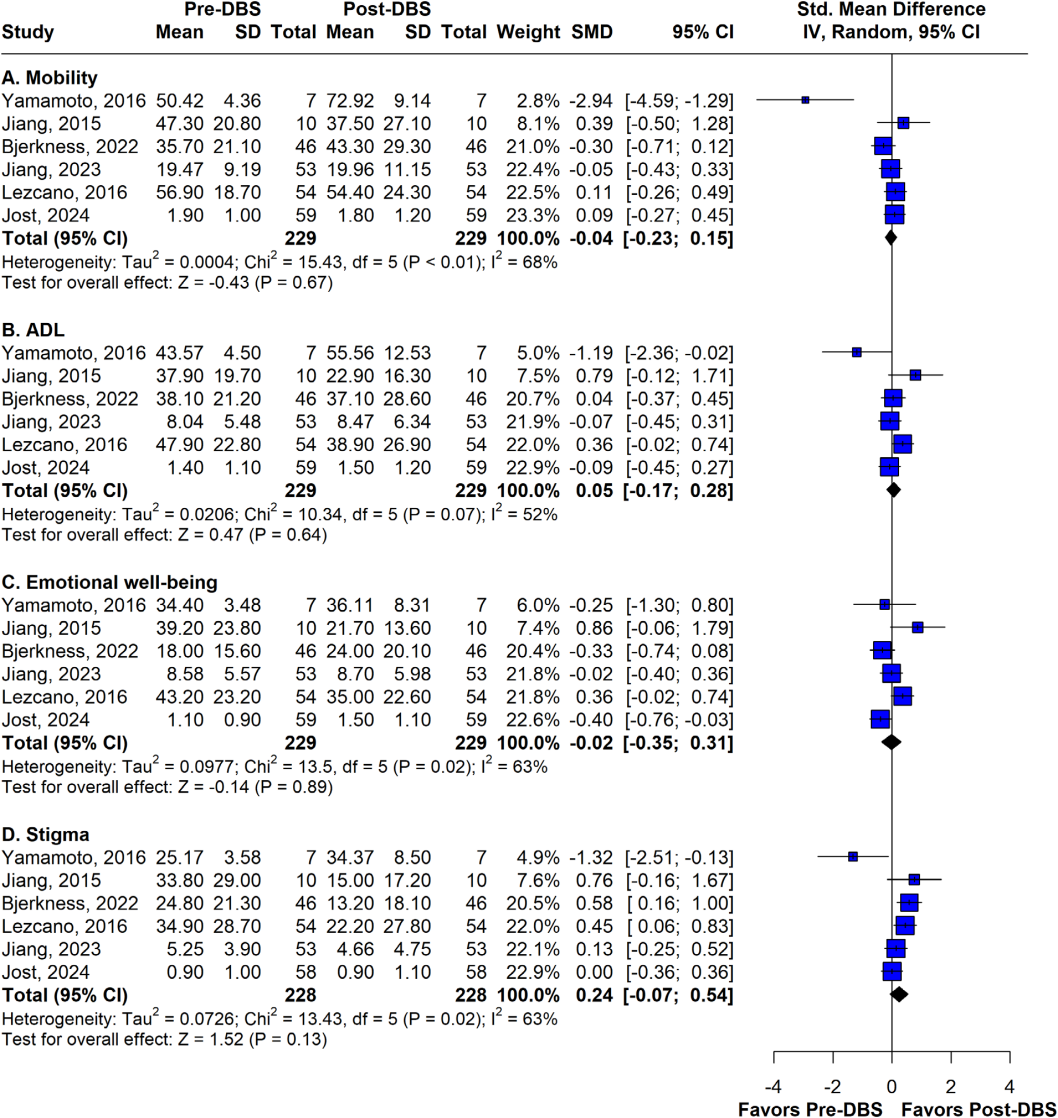
Forest plot of the mobility, ADL, emotional well‐being and stigma domains of PDQ at the 60 months follow‐up. ADL, activities of daily living; DBS, deep brain stimulation; SMD, standardized mean difference.

**Figure 4:**
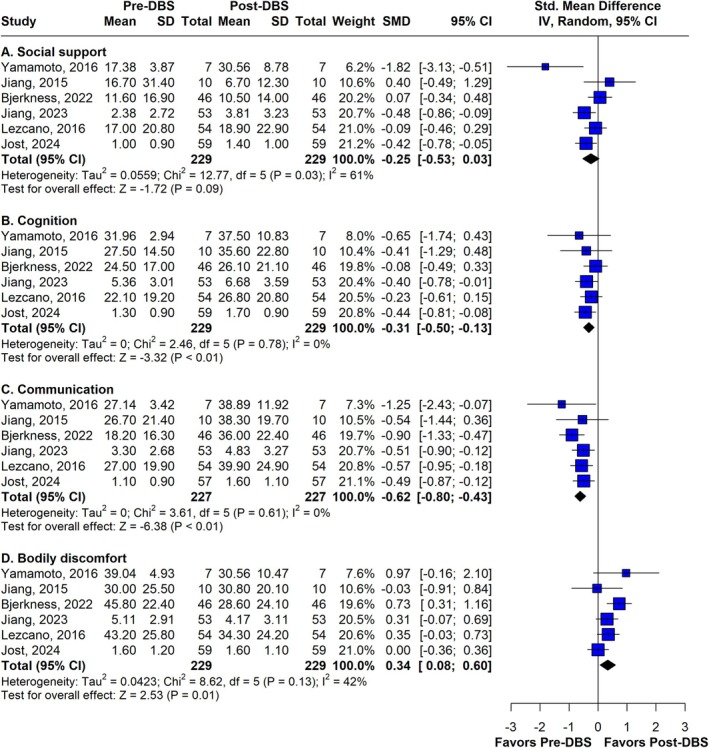
Forest plot of the social support, cognition, communication and bodily discomfort domains of PDQ at the 60 months follow‐up. DBS, deep brain stimulation; SMD, standardized mean difference.

In the 5‐year follow‐up, six studies evaluated each domain of the PDQ before and after surgery (*n* = 229). The meta‐analyses of these results indicated that the following domains returned to baseline values after DBS in PD patients: mobility, activities of daily living (ADL), emotional well‐being, stigma, and social support. The standardized mean differences (SMDs) were −0.04, 0.05, −0.02, 0.24, and −0.25, respectively. Additionally, some domains worsened over the period. Cognitive function significantly declined (SMD −0.31; 95% CI −0.50 to −0.13; I^2^ = 0%), and communication also deteriorated significantly (SMD −0.62; 95% CI −0.80 to −0.43; I^2^ = 0%). Bodily discomfort was the only domain to show a statistically significant improvement, although with a high heterogeneity (SMD 0.34; 95% CI 0.08 to 0.60; I^2^ = 42%). These analyses are presented in Figures 3 and 4.

### Meta‐Regression: Influence of Clinical Factors on Changes in QoL

Meta‐regression analyses at 12‐ and 24‐month follow‐ups found no significant associations for most factors. At 12 months, there were no associations for UPDRS Part II (*P* = 0.181), Part III (ON state) (*P* = 0.43), Part IV (*P* = 0.905), OFF‐to‐ON MDS‐UPDRS Part III change (*P* = 0.12), and LEDD (*P* = 0.09). Part III (OFF state) showed no effect at both 12 (*P* = 0.49) and 24 months (*P* = 0.346). Age at surgery (12 months: *P* = 0.065, 24 months: *P* = 0.652), disease duration (12 months: *P* = 0.266, 24 months: *P* = 0.195), and male proportion (12 months: *P* = 0.739, 24 months: *P* = 0.331) also had no significant impact on QoL.

Interestingly, baseline PDQ scores were a significant predictor of QoL improvement at 12 months (*P* = 0.011), with higher baseline PDQ values associated with greater QoL improvements (Fig. S2), although this effect was not observed at 24 months (*P* = 0.772) (Fig. S3).

### Subgroup Analysis: PDQ‐39

In comparison with the baseline score, the meta‐analyses showed an improvement in PDQ‐39 score of −11.8 points after 12 months, −11 points after 24 months, and – 5.7 points 36 months after surgery, followed by not significant deterioration of +0.6 score value after 60 months.

### Quality Assessment of the Included Studies

Figures S3 and S4 provide an overview of the risk of bias assessment using the RoB2 and ROBINS‐I tools to evaluate the quality of the studies included in this meta‐analysis. All RCTs were judged to have a low risk of bias. Among the nonrandomized studies, nearly all were rated as having a moderate risk of bias, with only one study assessed as having a serious risk.^24^ A funnel plot was generated for the meta‐analysis with the largest sample (12 months post‐surgery), revealing signs of asymmetry (Fig. S6), further supported by a significant Egger's test (*P* = 0.0003).

## Conclusion

This systematic review incorporated 6 to 34 bilateral STN‐DBS studies into multiple forest plots to examine the QoL trends in 208 to 1992 patients with PD. Key findings include: (1) patients experienced a significant improvement in QoL up to 36 months post‐surgery; (2) PDQ scores returned to pre‐operative levels by 60 months, indicating a lack of sustained long‐term benefits; (3) most PDQ domains reverted to baseline after five years, with cognitive function and communication notably deteriorating, although bodily discomfort showed a small, sustained improvement; (4) meta‐regression revealed no significant relationship between most clinical and sociodemographic factors at 12 and 24 months post‐surgery.

A threshold of −4.72 for detecting minimal clinically important improvement of PDQ‐39 scale has been previously established in a large study.^25^ As 38 out of the 42 included studies evaluated QoL through this scale, we were able to find in subgroup analysis that our results were not only statistically, but also clinically relevant until 36 months (improvements ranged between −5.7 and −11.8 at different follow‐ups). They also exceeded the improvement in PDQ‐39 achieved by oral therapy with administration of levodopa, catechol‐O‐methyltransferase inhibitors and dopamine agonists.^26^

Talking about other device‐aided therapies, apomorphine infusion showed PDQ improvement up to −13.2 points on a previous six‐month study,^27^ but a larger one found PDQ‐39 score was unchanged 24 months after treatment initiation.^28^ Intrajejunal infusion of levodopa–carbidopa intestinal gel improves PDQ‐39 up to −10 points,^29^ but current evidence has shown benefits only until 24 months on this scale.^30^ The lack of evidence on longevity of positive impact of these other invasive therapeutic approaches, in comparison with the clear long‐term benefit of STN‐DBS on QoL, is a critical consideration for those living with a chronic and disabling condition. It is important to note that patient populations receiving these therapies may differ from those undergoing STN‐DBS, especially in cases of significant neuropsychological impairment, where DBS may be advised against,^27^ among other contraindications.

Thus far, the only available RCTs show comparable benefits for HRQoL between STN and globus pallidus internus (GPi) targets for 3 years following DBS implantation.^31^, ^32^ However, in contrast to STN‐DBS, longer‐term outcomes of GPi‐DBS have rarely been studied,^33^ making QoL trend comparison between them unfeasible.

Consistent with a previous meta‐analysis,^7^ we also observed a decline in QoL measurements 60 months after bilateral STN‐DBS surgery. Although our analysis showed high heterogeneity (I^2^ = 45%), none of the seven included studies reported a sustained improvement in QoL after 5 years. Among these, only one study compared QoL outcomes between patients treated with STN‐DBS and those receiving standard‐of‐care medication, with the latter group showing significantly worse PDQ‐8 scores.^11^ We highlight that the return of QoL to baseline levels, observed in our analysis, might still represent a significant improvement compared with an optimized medication population. However, further studies directly comparing these groups are necessary to confirm these findings.

While motor symptoms remained improved across the included cohorts, it is possible that NMS, such as mood, cognition, cardio‐vascular, gastrointestinal and sexual manifestations contribute collectively to QoL deterioration over time. The NMS Scale, which helps identify specific NMS in PD and assess overall NMS burden,^34^ has a consistently reported strong link with PD HRQoL.^35^, ^36^ A meta‐analysis of ten studies^5^ indicated that the NMS Scale is significantly reduced by bilateral STN‐DBS; however, most included studies had limited follow‐up periods. Longer observational studies report varying results, with a tendency for the scale to return to preoperative levels within 4 years.^22^, ^37^ This aligns with the progression of NMS over time due to disease course, irrespective of dopaminergic replacement therapy.^38^, ^39^ Additionally, the development of L‐DOPA‐refractory or stimulation‐resistant axial problems, such as disturbances of gait, balance and speech become major determinants of QoL in the long term.^40^ These factors may explain the observed long‐term QoL trend, with a return to baseline levels as NMS and axial symptoms increase due to the progressive nature of PD.

To better understand the factors contributing to this regression in QoL, we further analyzed each domain of the PDQ at the 60‐month follow‐up. Notably, communication and cognition had significantly worsened (small and medium effect sizes, respectively) compared to the pre‐surgery assessment, making them key contributors to the observed QoL decline. Communication is assessed through questions addressing difficulties in speaking with others and in being understood by others. Our findings align with those of a previous cohort study,^41^ which identified a decline in speech intelligibility as a common adverse event of subthalamic stimulation, as well as the frequently observed decline in verbal fluency (both semantic and phonemic).^42^ Although bilateral cortical and subcortical damage is a possible result of both STN‐DBS surgery and lifetime brain stimulation, STN‐DBS does not seem to impose any additional deterioration of cognition in PD patients compared with medication therapy.^43^ Most probably, this cognitive deterioration is due to disease progression during these 60 months.

If one or more factors are consistently associated with postoperative PDQ outcomes, they should be evaluated during the DBS screening process and discussed with potential candidates. To our knowledge, this is the first meta‐analysis to assess the impact of various covariates on QoL up to 24 months post‐surgery using meta‐regression. Among the factors analyzed, a larger preoperative difference in motor scores between ON and OFF states was significantly associated with better postoperative QoL in a large study (n = 105),^44^ which used the Parkinson's Disease Quality of Life Questionnaire (PDQL), a scale with greater emphasis on motor aspects of QoL. In our analysis, which used the PDQ‐39 and PDQ‐8, no such correlation was found. A recent systematic review highlights the lack of consensus between studies and the unclear mechanisms linking these factors to postoperative QoL, suggesting that QoL improvements may be highly heterogeneous and individually determined, as well as scale dependent.^45^

The EARLY STIM trial demonstrated that STN‐DBS in PD should not be limited to patients in advanced stages, as patients with a mean age of 52 years and early motor complications experienced significant QoL improvements.^46^ This finding has been corroborated by more recent studies, which even included patients with earlier PD onset and those with known genetic mutations.^47^, ^48^ One study directly compared QoL outcomes between younger (mean age 52) and older patients (mean age 69), showing that STN‐DBS was equally effective in both matched cohorts.^49^ Notably, there is a lack of studies focusing on PD patients older than 70 years at the time of surgery. Since DBS candidate selection follows strict criteria, older patients or those with very advanced disease, who might experience worse outcomes, are largely excluded from studies.^49^ As seen in Table S1, this has resulted in little variability in mean age and disease duration across the included studies, which may help explain why these variables could not predict QoL in our meta‐regression analyses. Instead, assessing a patient's “physiological age,” which includes evaluations of frailty and comorbidities, may be more crucial in selecting suitable candidates for deep brain stimulation (DBS).^22^

In line with several short‐term studies,^50^, ^51^ our meta‐regression analysis found a potential link between higher mean baseline PDQ‐39 scores and greater postoperative QoL improvement, which could be partially explained by the regression‐to‐the‐mean phenomenon. In patients with exceptionally high baseline scores, the expectation is that their QoL can only improve or remain stable over time. Although this was not confirmed in the 24‐month meta‐regression, multivariate analysis from the EARLY STIM trial still identified baseline QoL as a predictor for improvements in this period.^52^ Additionally, another study supported baseline QoL as a predictor for QoL improvements up to 36 months.^22^

This study has several limitations. First, significant heterogeneity in QoL scores across all follow‐up periods may raise questions about the reliability of our findings. This variability could be attributed to differences in sociodemographic and clinical factors, variations in DBS techniques, and even differences in anesthesia protocols. However, the long‐term trend in QoL was clearly defined, showing a return to preoperative levels, with some domains even worsening. Second, the studies with 36‐ and 60‐month follow‐ups experienced substantial participant attrition, which could have introduced bias due to missing data. Third, the asymmetric funnel plot observed at the 12‐month follow‐up suggests a potential overestimation of the short‐term effect of STN‐DBS on QoL. This pattern may be attributable to publication bias (ie, the tendency for smaller studies to be published only when they present positive findings). A significant limitation of a previous meta‐analysis evaluating QoL after STN‐DBS is that this important source of bias was not assessed.^7^ Fourth, the predominance of male participants in the studies included in this review highlights the underrepresentation of women in DBS research,^53^ which hinders the identification of sex‐specific therapeutic nuances. Finally, the analysis of covariates using meta‐regression is exploratory, and no definitive conclusions can be drawn. Nonetheless, we emphasize the need for future studies to explore the correlation between baseline PDQ scores and postoperative improvements following STN‐DBS, as subjectively relevant changes in QoL could be considered, at least in the short term, as a potential predictive factor.

This meta‐analysis of 2767 PD patients who underwent bilateral STN‐DBS, accurately identified long‐term QoL trend, showing improvement in QoL as measured by the PDQ up to 36 months postoperatively, followed by a decline back to preoperative levels after 60 months, with NMS and axial features playing a significant role in this trend. Future studies should aim to identify other factors contributing to this loss of beneficial effects in long‐term QoL which we observed across nearly all PDQ domains. Our analysis of various covariates revealed that the available data are insufficient to suggest changes in clinical practice for identifying candidates who are more likely to experience sustained QoL improvements after DBS surgery. More robust multivariate models are still needed to refine patient selection criteria.

## Author Roles

(1) Research project: A. Conception, B. Organization, C. Execution; (2) Statistical Analysis: A. Design, B. Execution, C. Review and Critique; (3) Manuscript: A. Writing of the first draft, B. Review and Critique.

L.O.N.: 1A, 1C, 2C, 3A, 3B

A.M.S.C.: 1B, 1C

L.D.C.D.: 1C, 3A

V.L.J.A.: 2A, 2B, 2C

M.M.F.: 1B, 3A

I.S.M.R.: 1B, 1C

M.V.D.C.: 2C, 3A

C.C.S.: 3A, 3B

L.B.B.:1B, 3B

H.B.F.: 2C, 3B

R.L.O.A.: 2C, 3B

D.L.B.: 1A, 1B, 1C, 3A

## Disclosures

**Ethical Compliance Statement:** The authors confirm that the approval of an institutional review board and informed patient consent was not required for this work. We confirm that we have read the Journal's position on issues involved in ethical publication and affirm that this work is consistent with those guidelines.

**Funding Sources and Conflict of Interest:** No specific funding was received for this work. The authors declare that there are no conflicts of interest relevant to this work.

**Financial Disclosures for the Previous 12 Months:** The authors declare that they have no financial disclosures to report for the previous 12 months.

## Supporting information


**Supplementary TABLE S1.** Baseline characteristics of included studies.
**Supplementary Figure S1.** Forest plot of the follow‐up period of 12 months showing the SMD and the weights for the random effects analysis of each study. DBS, deep brain stimulation; SMD, standardized mean difference.
**Supplementary Figure S2.** Meta‐regression modeling for the impact of mean baseline PDQ score and QoL at 12 months follow‐up (*P* = 0.011).
**Supplementary Figure S3.** Meta‐regression modeling for the impact of mean baseline PDQ score and QoL at 24 months follow‐up (*P* = 0.772).
**Supplementary Figure S4.** Critical appraisal of individual studies according to Cochrane's tools for assessing risk of bias in non‐randomized studies (ROBINS‐I).
**Supplementary Figure S5.** Critical appraisal of individual studies according to the Cochrane Collaboration's tool for assessing risk of bias in randomized trials (RoB 2).
**Supplementary Figure S6.** Funnel plot of the follow‐up period of 12 months showing signs of asymmetry, indicating small‐study effect, confirmed by Egger's test (*P* = 0.0003).

## Data Availability

The data that supports the findings of this study are available in the supplementary material of this article.
