# Supplementary material for: Long‐Term Quality of Life Trend after Subthalamic Stimulation for Parkinson's Disease: An Updated Systematic Review and Meta‐Analysis
Source: Mov Disord Clin Pract. 2025 Mar 5;12(5):577–87. doi: 10.1002/mdc3.70025 (PMC12070175; doi:10.1002/mdc3.70025)

Supplementary Table 1. Baseline characteristics of included studies

Study (Author, year)	Sample Size (n)	Baseline PDQ Score (mean ± SD)	Months of Follow-Up	Follow-up PDQ Score (mean ± SD)	Mean Age at Surgery (years)	Disease Duration at Surgery (years)	Gender (% Male)
Acera 2019	40	38.1 ± 13	12<br>60	27.4 ± 14.6<br>36.7 ± 15.4	62.2	14.1	64
Bjerkness 2020	53	27 ± 12	12	20 ± 15	62	11	75
Bjerkness 2022	46	26 ± 11.9	60	27.3 ± 15.2	63	12	72
Boussac 2021	303	31.3 ± 11.4	12	26.8 ± 13.5	60	10.1	67.3
Chan 2016	18	47.1 ± 10.9	12<br>24	32.1 ± 11.4<br>28.3 ± 11.6	55	13	68
Chen asleep 2023<br>Chen awake 2023	25<br>21	29.2 ± 12.1<br>29.4 ± 11.7	12<br>12	21.8 ± 14.3<br>21.1 ± 12.2	57.3<br>53.8	10.3<br>9.0	68<br>71
Chircop 2018	24	37.5 ± 14.7	12	17.9 ± 9.5	60.2	8.8	62
Dafsari 2018	65	33.3 ± 17.4*	24	30.6 ± 18.5*	62.3	10.9	75
Drapier 2005	27	44.1 ± 10.9	12	34.8 ± 12.3	60.8	14.6	70
Erdem 2023	22	42.3 ± 22	12	27.1 ± 22	57.3	11	63.6
Follett 2010	147	46.9 ± 12.6	24	42.7 ± 15.6	61.9	11.1	79
Georgiev 2021	28	53.4 ± 4.5	12<br>24<br>36	39.0 ± 3.6<br>40.2 ± 2.9<br>46.14 ± 4.1	64.4	8.5	61
Hong 2024	27	28.9 ± 9.2	24	17.6 ± 3.7	48.4	8.9	67
Houeto 2006	20	43.1 ± 13.1	24	30.9 ± 13	54.9	10.9	75
Jiang 2015	10	32.4 ± 14.1	12<br>36<br>60	19.8 ± 8.5<br>13.5 ± 10.5<br>26.1 ± 9.7	59.4	9.3	60
Jiang 2023	53	36.73 ± 16.86	12<br>60	27.29 ± 16.51<br>38.8 ± 21.19	58.89	9.45	45
Jost 2021	73	32.8 ± 16.8*	36	31.1 ± 20.2*	62	10.3	59
Jost 2024	61<br>57	31.8 ± 14.5*<br>31.8 ± 14.5*	12<br>60	23.4 ± 14.6*<br>37.1 ± 17.0*	62.6	10.4	60
Krause 2022	46	39 ± 15	12	28.8 ±18	51	12.9	59
Lewis 2014	28	36.3 ± 13.76	12	30.84 ±14.07	61.18	12.43	61
Lezcano 2016	64<br>54	41.1 ± 14.1<br>41.1 ± 14.1	12<br>60	26.1 ± 14.1<br>37.5 ± 16.5	61.3<br>61.1	13.2<br>13	61<br>57

Li 2020	16	52.49 ± 17.93	12	24.72 ± 9.83	60.25	10.38	50
Lin 2021	40	63.20 ± 26.43	12	45.20 ± 29.74	61.3	9.98	57
Liu 2019	45	39.05 ± 14.23	12	31.45 ± 17.75	61.8	11.9	47
Liu 2021	47<br>23	34.00 ± 2.64	12<br>36	23.45 ± 2.45<br>31.68 ± 3.44	65.3	11.9	n.r.
Lu 2020	87	58 ± 1.92	12	34.74 ± 5	62	n.r.	70
Lu_LA 2022<br><br>Lu_GA 2022	81<br><br>76	64 ± 9.6<br><br>64.7 ± 7.8	12<br>24<br>12<br>24	40.6 ± 5.7<br>48.0 ± 5.1<br>42.4 ± 9.1<br>48.7 ± 7.8	63.7<br><br>62.8	9.5<br><br>9.3	60<br><br>60
Luo 2023	12	33.0 ± 10.3	12	20.9 ± 14.1	61.3	11.3	50
Lyons 2005	59	41.7 ± 11.8	12	28.2 ± 13.6	59.5	11.9	75
Mameli 2022	20	40.15 ± 14.80*	12	30.73 ± 17.88*	57.6	10.95	40
Moran 2020	137	37.59 ± 21.15	12	29.98 ± 23.31	60	11	n.r.
Nazzaro 2011	24	32.4 ± 14.2	12	17.2 ± 9.6	64.2	10.6	67
Pintér direc. 2022<br>Pintér omni. 2022	52<br>57	28.4 ± 15<br>24.5 ± 12.2	12<br>12	17.3 ± 10.8<br>18.9 ± 13.9	60.3<br>59.7	9.7<br>10.3	63<br>65
Schuepbach 2013	110	30.1 ± 14.68	24	22 ± 12.59	52.9	7.3	76
Sobstyl 2014	16<br>14	35.4 ± 6.6<br>35.8 ± 6.9	12<br>24	24.7 ± 4.2<br>26.0 ± 3.9	63.5	n.r.	69
Soulas 2011	35	49.89 ± 11.06	12	39.12 ± 16.09	62.02	14.49	n.r.
Tykocki 2012	74	64.95 ± 30.09	12<br>24	39.51 ± 17.54<br>43.15 ± 20.54	55.6	12.3	51
Vats young 2019<br><br>Vats old 2019	20<br><br>20	24.55 ± 2.14<br><br>22.82 ± 3.33	12<br>24<br>12<br>24	19.59 ± 2.32<br>17.54 ± 2.51<br>19.90 ± 1.95<br>18.16 ± 1.84	51.95<br><br>68.75	n.r.	75
Vinke 2022	29	46.9 ± 21.5	12	39.2 ± 20.8	62	9	83
Weaver 2012	66<br>62	48.1 ± 13.2	24<br>36	39.5 ± 15.3<br>44 ± 15.1	60.7	11.3	80
Williams 2010	183	37.5 ± 14.6	12	32.5 ± 15.8	59	11.5	68
Yamamoto 2016	31<br>12<br>7	33.63 ± 2.70<br>33.63 ± 2.70<br>33.63 ± 2.70	12<br>36<br>60	28.29 ± 2.72<br>28.08 ± 5.61<br>42.05 ± 7.77	66.7	11.6	n.r.

* PDQ8 was used to measure quality of life.
n.r., not reported; PDQ, Parkinson's Disease Questionnaire.

REFERENCES OF INCLUDED STUDIES

1. Acera M, Molano A, Tijero B, et al. Long-term impact of subthalamic stimulation on cognitive function in patients with advanced Parkinson's disease. Neurologia (Engl Ed). 2019 Nov-Dec;34(9):573-581. English, Spanish. doi: 10.1016/j.nrl.2017.05.009. Epub 2017 Jul 14
2. Bjerknes S, Skogseid IM, Hauge TJ, et al. Subthalamic deep brain stimulation improves sleep and excessive sweating in Parkinson's disease. NPJ Parkinsons Dis. 2020 Oct 14;6:29. doi: 10.1038/s41531-020-00131-0
3. Bjerknes S, Toft M, Brandt R, et al. Subthalamic Nucleus Stimulation in Parkinson's Disease: 5-Year Extension Study of a Randomized Trial. Mov Disord Clin Pract. 2021 Oct 18;9(1):48-59. doi: 10.1002/mdc3.13348
4. Boussac M, Arbus C, Klinger H, et al. Personality Related to Quality-of-Life Improvement After Deep Brain Stimulation in Parkinson's Disease (PSYCHO-STIM II). *J Parkinsons Dis*. 2022;12(2):699-711. doi:10.3233/JPD-212883
5. Chan DT, Zhu CX, Lau CK, et al. Subthalamic Nucleus Deep Brain Stimulation for Parkinson Disease in Hong Kong: A Prospective Territory-Wide 2-Year Follow-Up Study. *World Neurosurg*. 2016;93:229-236. doi:10.1016/j.wneu.2016.06.002.
6. Chen W, Zhang C, Jiang N, et al. The efficacy and safety of asleep and awake subthalamic deep brain stimulation for Parkinson's disease patients: A 1-year follow-up. *Front Aging Neurosci*. 2023;15:1120468. Published 2023 Apr 18. doi:10.3389/fnagi.2023.1120468
7. Chircop C, Dingli N, Aquilina A, Zrinzo L, Aquilina J. MRI-verified "asleep" deep brain stimulation in Malta through cross border collaboration: clinical outcome of the first five years. *Br J Neurosurg*. 2018;32(4):365-371. doi:10.1080/02688697.2018.1478061
8. Dafsari HS, Silverdale M, Strack M, et al. Nonmotor symptoms evolution during 24 months of bilateral subthalamic stimulation in Parkinson's disease. *Mov Disord*. 2018;33(3):421-430. doi:10.1002/mds.27283
9. Drapier S, Raoul S, Drapier D, et al. Only physical aspects of quality of life are significantly improved by bilateral subthalamic stimulation in Parkinson's disease. *J Neurol*. 2005;252(5):583-588. doi:10.1007/s00415-005-0704-4
10. Erdem NŞ, Gencer GYG, Özkaynak SS, Uçar T, Baysal ÖD. Neuropsychiatric Effects of Bilateral Subthalamic Nucleus Deep Brain Stimulation in Parkinson's Disease: Results at the 12-Month Follow-up. *Noro Psikiyatr Ars*. 2023;60(2):169-173. Published 2023 May 4. doi:10.29399/npa.28241
11. Follett KA, Weaver FM, Stern M, et al. Pallidal versus subthalamic deep-brain stimulation for Parkinson's disease. *N Engl J Med*. 2010;362(22):2077-2091. doi:10.1056/NEJMoa0907083
12. Georgiev D, Mencinger M, Rajnar R, et al. Long-term effect of bilateral STN-DBS on non-motor symptoms in Parkinson's disease: A four-year observational, prospective study. *Parkinsonism Relat Disord*. 2021;89:13-16. doi:10.1016/j.parkreldis.2021.06.017
13. Hong J, Xie H, Chen Y, et al. Effects of STN-DBS on cognition and mood in young-onset Parkinson's disease: a two-year follow-up. *Front Aging Neurosci*. 2024;15:1177889. Published 2024 Jan 16. doi:10.3389/fnagi.2023.1177889
14. Houeto JL, Mallet L, Mesnage V, et al. Subthalamic stimulation in Parkinson disease: behavior and social adaptation. *Arch Neurol*. 2006;63(8):1090-1095. doi:10.1001/archneur.63.8.1090
15. Jiang LL, Liu JL, Fu XL, et al. Long-term Efficacy of Subthalamic Nucleus Deep Brain Stimulation in Parkinson's Disease: A 5-year Follow-up Study in China. *Chin Med J (Engl)*. 2015;128(18):2433-2438. doi:10.4103/0366-6999.164925
16. Jiang JL, Chen SY, Tsai ST, Ma YC, Wang JH. Long-Term Effects of Subthalamic Stimulation on Motor Symptoms and Quality of Life in Patients with Parkinson's Disease. *Healthcare (Basel)*. 2023;11(6):920. Published 2023 Mar 22. doi:10.3390/healthcare11060920
17. Jost ST, Visser-Vandewalle V, Rizos A, et al. Non-motor predictors of 36-month quality of life after subthalamic stimulation in Parkinson disease. *NPJ Parkinsons Dis*. 2021;7(1):48. Published 2021 Jun 8. doi:10.1038/s41531-021-00174-x
18. Jost ST, Aloui S, Evans J, et al. Neurostimulation for Advanced Parkinson Disease and Quality of Life at 5 Years: A Nonrandomized Controlled Trial. *JAMA Netw Open*. 2024;7(1):e2352177. Published 2024 Jan 2. doi:10.1001/jamanetworkopen.2023.52177
19. Krause P, Reimer J, Kaplan J, et al. Deep brain stimulation in Early Onset Parkinson's disease. *Front Neurol*. 2022;13:1041449. Published 2022 Nov 17. doi:10.3389/fneur.2022.1041449
20. Lewis CJ, Maier F, Eggers C, et al. Parkinson's disease patients with subthalamic stimulation and carers judge quality of life differently. *Parkinsonism Relat Disord*. 2014;20(5):514-519. doi:10.1016/j.parkreldis.2014.02.009
21. Lezcano E, Gómez-Esteban JC, Tijero B, et al. Long-term impact on quality of life of subthalamic nucleus stimulation in Parkinson's disease. *J Neurol*. 2016;263(5):895-905. doi:10.1007/s00415-016-8077-4
22. Li H, Liang S, Yu Y, et al. Effect of Subthalamic Nucleus Deep Brain Stimulation (STN-DBS) on balance performance in Parkinson's disease. *PLoS One*. 2020;15(9):e0238936. Published 2020 Sep 11. doi:10.1371/journal.pone.0238936

23. Lin W, Shi D, Wang D, Yang L, Wang Y, Jin L. Can Levodopa Challenge Testing Predict the Effect of Deep Brain Stimulation? One-Year Outcomes in a Chinese Cohort. *Front Aging Neurosci*. 2021;13:764308. Published 2021 Oct 20. doi:10.3389/fnagi.2021.764308
24. Liu FT, Lang LQ, Yang YJ, et al. Predictors to quality of life improvements after subthalamic stimulation in Parkinson's disease. *Acta Neurol Scand*. 2019;139(4):346-352. doi:10.1111/ane.13056
25. Liu W, Yamamoto T, Yamanaka Y, et al. Neuropsychiatric Symptoms in Parkinson's Disease After Subthalamic Nucleus Deep Brain Stimulation. *Front Neurol*. 2021;12:656041. Published 2021 May 4. doi:10.3389/fneur.2021.656041
26. Jingchao Lu, Zhaohai Feng, Xin Shi, Lei Jiang, Yujun Hao, Correlation between programmed stimulation parameters and their efficacy after deep brain electrode implantation for Parkinson's disease, Journal of Neurorestoratology, Volume 8, Issue 1, 2020, Pages 53-59. https://doi.org/10.26599/JNR.2019.9040018.
27. Lu Y, Chang L, Li J, et al. The Effects of Different Anesthesia Methods on the Treatment of Parkinson's Disease by Bilateral Deep Brain Stimulation of the Subthalamic Nucleus. *Front Neurosci*. 2022;16:917752. Published 2022 May 26. doi:10.3389/fnins.2022.917752
28. Luo G, Shi X, Jiang L, et al. Effects of STN-DBS surgery on cerebral glucose metabolism and distribution of DAT in Parkinson's disease. *Brain Behav*. 2023;13(8):e3172. doi:10.1002/brb3.3172
29. Lyons KE, Pahwa R. Long-term benefits in quality of life provided by bilateral subthalamic stimulation in patients with Parkinson disease. *J Neurosurg*. 2005;103(2):252-255. doi:10.3171/jns.2005.103.2.0252
30. Mameli F, Ruggiero F, Dini M, et al. Energy Delivered by Subthalamic Deep Brain Stimulation for Parkinson Disease Correlates With Depressive Personality Trait Shift. *Neuromodulation*. 2023;26(2):394-402. doi:10.1016/j.neurom.2022.01.004
31. Moran CH, Pietrzyk M, Sarangmat N, et al. Clinical Outcome of "Asleep" Deep Brain Stimulation for Parkinson Disease Using Robot-Assisted Delivery and Anatomic Targeting of the Subthalamic Nucleus: A Series of 152 Patients. *Neurosurgery*. 2020;88(1):165-173. doi:10.1093/neuros/nyaa367
32. Nazzaro JM, Pahwa R, Lyons KE. The impact of bilateral subthalamic stimulation on non-motor symptoms of Parkinson's disease. *Parkinsonism Relat Disord*. 2011;17(8):606-609. doi:10.1016/j.parkreldis.2011.05.009
33. Pintér D, Járdaházi E, Balás I, et al. Antiparkinsonian Drug Reduction After Directional Versus Omnidirectional Bilateral Subthalamic Deep Brain Stimulation. *Neuromodulation*. 2023;26(2):374-381. doi:10.1016/j.neurom.2022.01.006
34. Schuepbach WM, Rau J, Knudsen K, et al. Neurostimulation for Parkinson's disease with early motor complications. *N Engl J Med*. 2013;368(7):610-622. doi:10.1056/NEJMoa1205158
35. Sobstyl M, Ząbek M, Górecki W, Mossakowski Z. Quality of life in advanced Parkinson's disease after bilateral subthalamic stimulation: 2 years follow-up study. *Clin Neurol Neurosurg*. 2014;124:161-165. doi:10.1016/j.clineuro.2014.06.019
36. Soulas T, Sultan S, Gurruchaga JM, Palfi S, Fénelon G. Depression and coping as predictors of change after deep brain stimulation in Parkinson's disease. *World Neurosurg*. 2011;75(3-4):525-532. doi:10.1016/j.wneu.2010.06.015
37. Tykocki T, Szalecki K, Koziara H, Nauman P, Mandat T. Quality of life and depressive symptoms in Parkinson's disease after subthalamic deep brain stimulation: a 2-year follow-up study. *Turk Neurosurg*. 2013;23(3):379-384. doi:10.5137/1019-5149.JTN.7184-12.1
38. Vats A, Amit A, Doshi P. A comparative study of bilateral subthalamic nucleus DBS in Parkinson's disease in young versus old: A single institutional study. *J Clin Neurosci*. 2019;70:85-91. doi:10.1016/j.jocn.2019.08.065
39. Vinke RS, Selvaraj AK, Geerlings M, et al. The Role of Microelectrode Recording and Stereotactic Computed Tomography in Verifying Lead Placement During Awake MRI-Guided Subthalamic Nucleus Deep Brain Stimulation for Parkinson's Disease. *J Parkinsons Dis*. 2022;12(4):1269-1278. doi:10.3233/JPD-223149
40. Weaver FM, Follett KA, Stern M, et al. Randomized trial of deep brain stimulation for Parkinson disease: thirty-six-month outcomes. *Neurology*. 2012;79(1):55-65. doi:10.1212/WNL.0b013e31825dcdc1
41. Williams A, Gill S, Varma T, et al. Deep brain stimulation plus best medical therapy versus best medical therapy alone for advanced Parkinson's disease (PD SURG trial): a randomised, open-label trial. *Lancet Neurol*. 2010;9(6):581-591. doi:10.1016/S1474-4422(10)70093-4
42. Yamamoto T, Uchiyama T, Higuchi Y, et al. Long term follow-up on quality of life and its relationship to motor and cognitive functions in Parkinson's disease after deep brain stimulation. *J Neurol Sci*. 2017;379:18-21. doi:10.1016/j.jns.2017.05.037

Supplementary Figure 1. Forest plot of the follow-up period of 12 months showing the SMD and the weights for the random effects analysis of each study. DBS, deep brain stimulation; SMD, standardized mean difference.

Study	Pre-DBS Mean	Pre-DBS SD	Pre-DBS Total	Post-DBS Mean	Post-DBS SD	Post-DBS Total	Weight	SMD	95% CI
**12 months follow-up**									
Jiang, 2015	32.40	14.10	10	19.80	8.50	10	2.4%	1.04	[0.09; 1.98]
Luo, 2023	33.00	10.30	12	20.90	14.10	12	2.4%	0.95	[0.09; 1.80]
Sobstyl, 2014	35.40	6.60	16	24.70	4.20	16	2.4%	1.89	[1.04; 2.74]
Georgiev, 2021	53.40	4.50	28	39.00	3.60	28	2.5%	3.48	[2.64; 4.33]
Li, 2020	52.49	17.93	16	24.72	9.83	16	2.5%	1.87	[1.02; 2.72]
Vats_young, 2019	24.55	2.14	20	19.59	2.32	20	2.5%	2.18	[1.38; 2.98]
Chan, 2016	47.10	10.90	18	32.10	11.40	18	2.5%	1.32	[0.59; 2.04]
Liu, 2021	34.00	2.64	47	23.45	2.45	47	2.5%	4.11	[3.39; 4.83]
Lu, 2020	58.00	1.92	87	34.74	5.00	87	2.5%	6.11	[5.40; 6.83]
Vats_old, 2019	22.82	3.33	20	19.90	1.95	20	2.6%	1.05	[0.38; 1.71]
Chircop, 2018	37.50	14.70	24	17.90	9.50	24	2.6%	1.56	[0.91; 2.21]
Mameli, 2022	40.15	14.80	20	30.73	17.88	20	2.6%	0.56	[-0.07; 1.20]
Chen_awake, 2023	29.40	11.70	21	21.17	12.21	21	2.6%	0.68	[0.05; 1.30]
Nazzaro, 2011	32.40	14.20	24	17.20	9.60	24	2.6%	1.23	[0.61; 1.85]
Yamamoto, 2016	33.63	2.70	31	28.29	2.72	31	2.6%	1.95	[1.34; 2.56]
Erdem, 2023	42.30	22.00	22	27.10	22.00	22	2.6%	0.68	[0.07; 1.29]
Chen_asleep, 2023	29.26	12.07	25	21.86	14.35	25	2.6%	0.55	[-0.02; 1.11]
Drapier, 2005	44.10	10.90	27	34.80	12.30	27	2.6%	0.79	[0.23; 1.34]
Lewis, 2014	36.30	13.76	28	30.84	14.07	28	2.7%	0.39	[-0.14; 0.92]
Vinke, 2022	46.90	21.50	29	39.20	20.80	29	2.7%	0.36	[-0.16; 0.88]
Soulas, 2011	49.89	11.06	35	39.12	16.09	35	2.7%	0.77	[0.28; 1.26]
Lin, 2021	63.20	26.43	40	45.20	29.74	40	2.7%	0.63	[0.18; 1.08]
Lu_LA, 2022	64.00	9.60	81	40.60	5.70	81	2.7%	2.95	[2.50; 3.40]
Lu_GA, 2022	64.70	7.80	76	42.40	9.10	76	2.7%	2.62	[2.18; 3.05]
Liu, 2019	39.05	14.23	45	31.45	17.75	45	2.7%	0.47	[0.05; 0.89]
Krause, 2022	39.00	15.00	46	28.80	18.00	46	2.7%	0.61	[0.19; 1.03]
Acera, 2019	38.10	13.03	48	27.46	14.61	48	2.7%	0.76	[0.35; 1.18]
Pintér direc., 2022	28.40	15.00	52	17.30	10.80	52	2.7%	0.84	[0.44; 1.24]
Jiang, 2023	36.73	16.86	53	27.29	16.51	53	2.7%	0.56	[0.17; 0.95]
Bjerkness, 2020	27.00	12.00	53	20.00	15.00	53	2.7%	0.51	[0.12; 0.90]
Lyons, 2005	41.70	11.80	59	28.20	13.60	59	2.7%	1.05	[0.67; 1.44]
Pintér omni., 2022	24.50	12.20	57	18.90	13.90	57	2.7%	0.43	[0.05; 0.80]
Lezcano, 2016	41.10	14.10	64	26.10	14.10	64	2.7%	1.06	[0.69; 1.43]
Jost, 2024	31.80	14.50	61	23.40	14.60	61	2.7%	0.57	[0.21; 0.94]
Tykock, 2012	64.95	30.09	74	39.51	17.54	74	2.7%	1.03	[0.68; 1.37]
Moran, 2020	37.59	21.15	137	29.98	23.31	137	2.8%	0.34	[0.10; 0.58]
Williams, 2010	37.50	14.60	183	32.50	15.80	183	2.8%	0.33	[0.12; 0.53]
Boussac, 2021	31.30	11.40	303	26.80	13.50	303	2.8%	0.36	[0.20; 0.52]
**Total (95% CI)**			**1992**			**1992**	**100.0%**	**1.26**	**[0.88; 1.63]**

Heterogeneity: $Tau^2 = 1.3191$; $Chi^2 = 621.58$, df = 37 ($P < 0.01$); $I^2 = 94\%$
Test for overall effect: $Z = 6.55$ ($P < 0.01$)

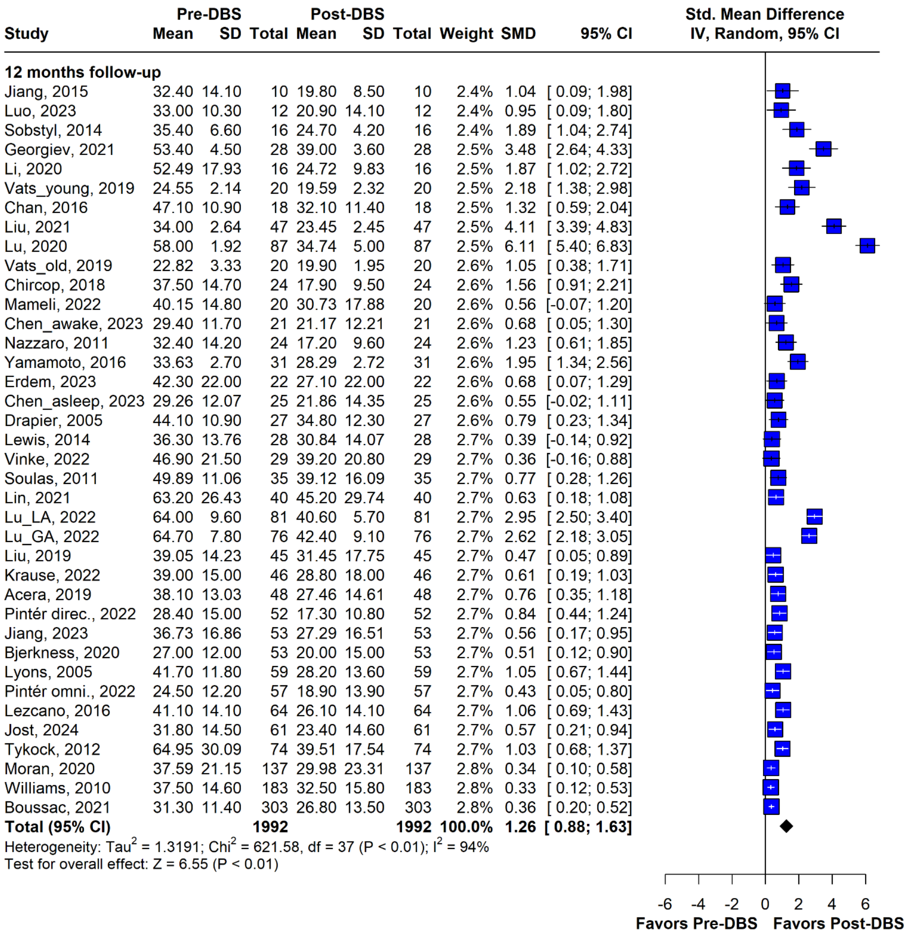

Supplementary Figure 2. Meta-regression modeling for the impact of mean baseline PDQ score and QoL at 12 months follow-up (p = 0.011).

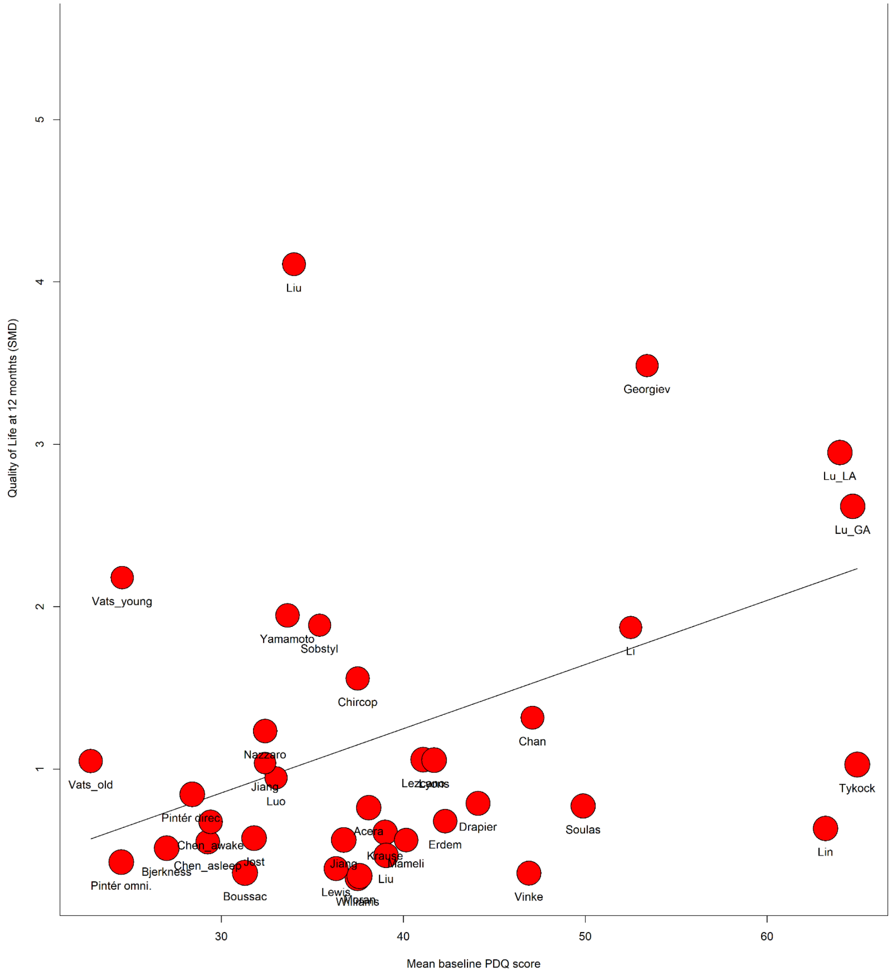

Supplementary Figure 3. Meta-regression modeling for the impact of mean baseline PDQ score and QoL at 24 months follow-up (p = 0.772).

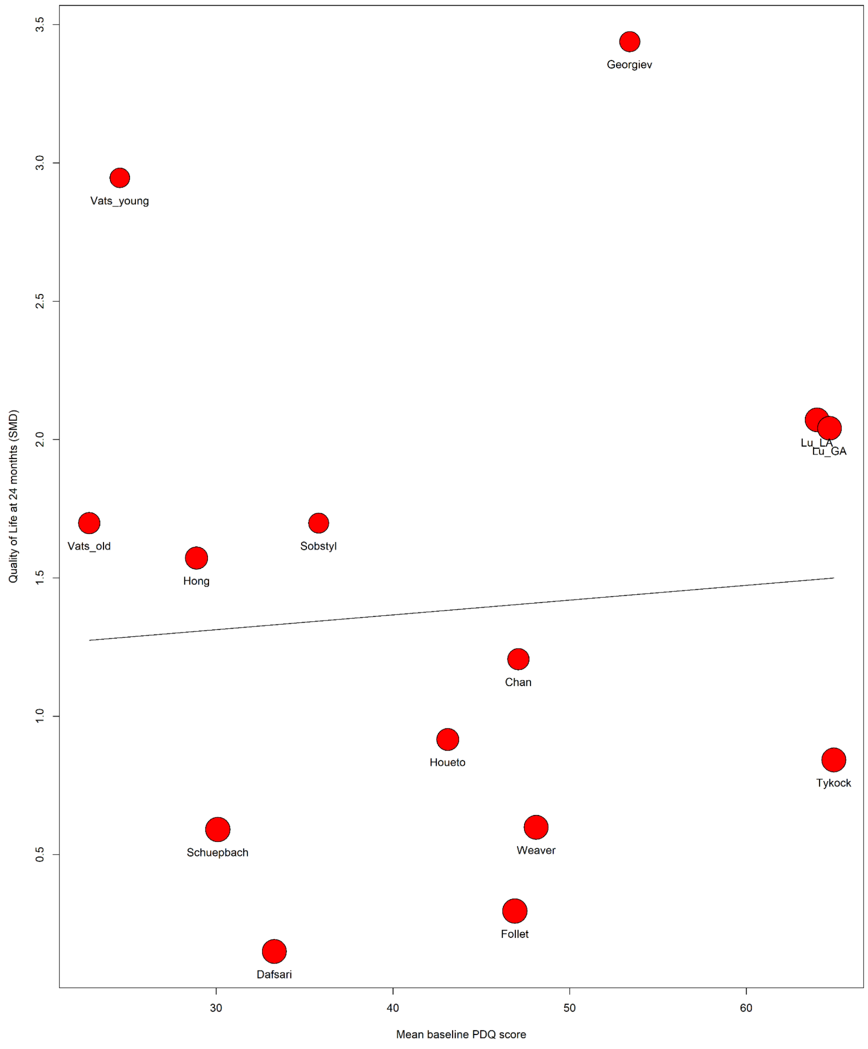

Supplementary Figure 4. Critical appraisal of individual studies according to Cochrane's tools for assessing risk of bias in non-randomized studies (ROBINS-I).

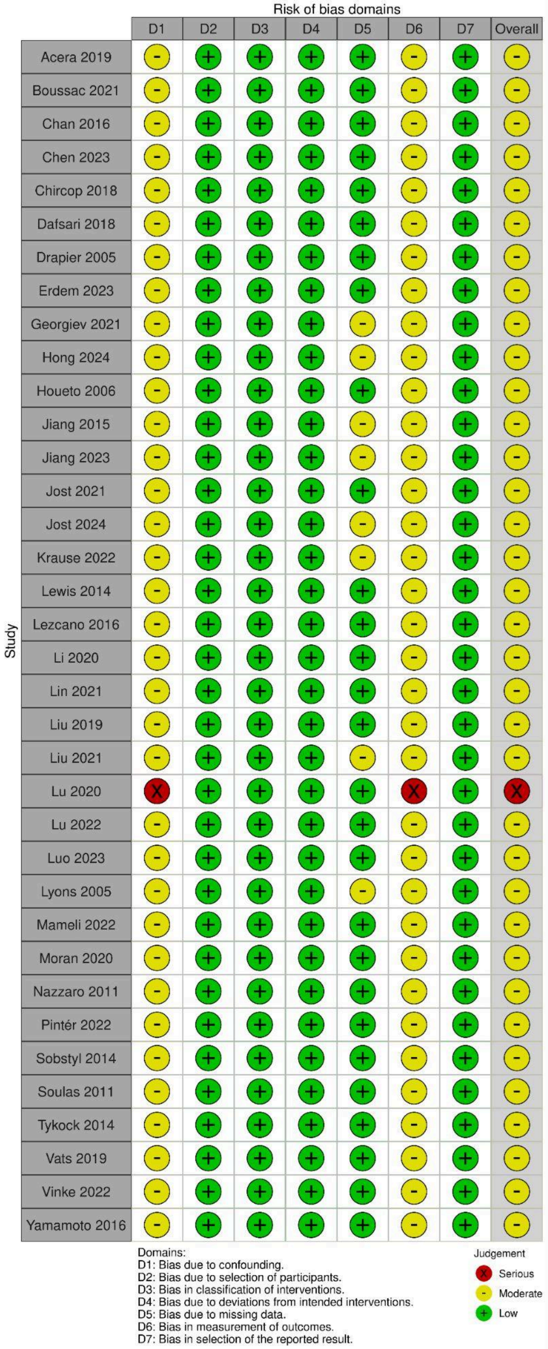

Supplementary Figure 5. Critical appraisal of individual studies according to the Cochrane's Collaboration's tool for assessing risk of bias in randomized trials (RoB 2).

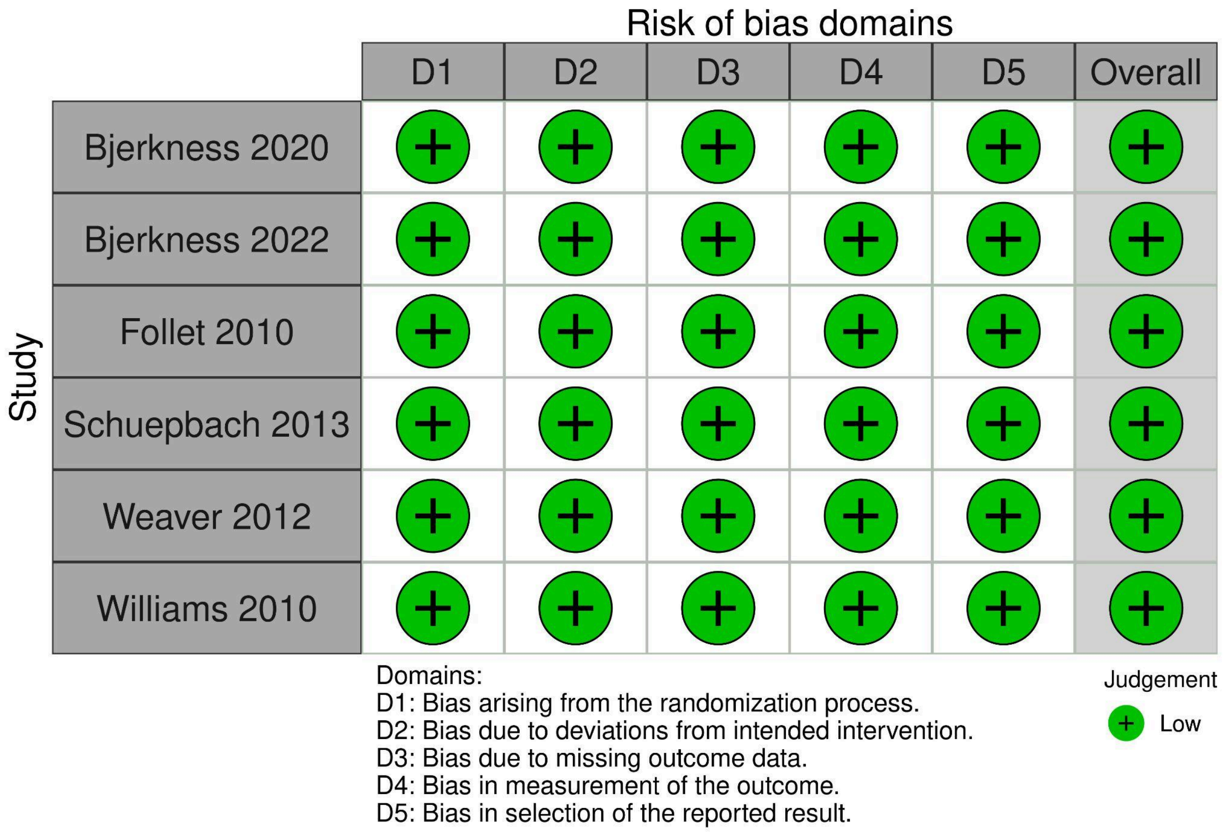

Supplementary Figure 6. Funnel plot of the follow-up period of 12 months showing signs of asymmetry, indicating small-study effect, confirmed by Egger's test (p=0.0003).

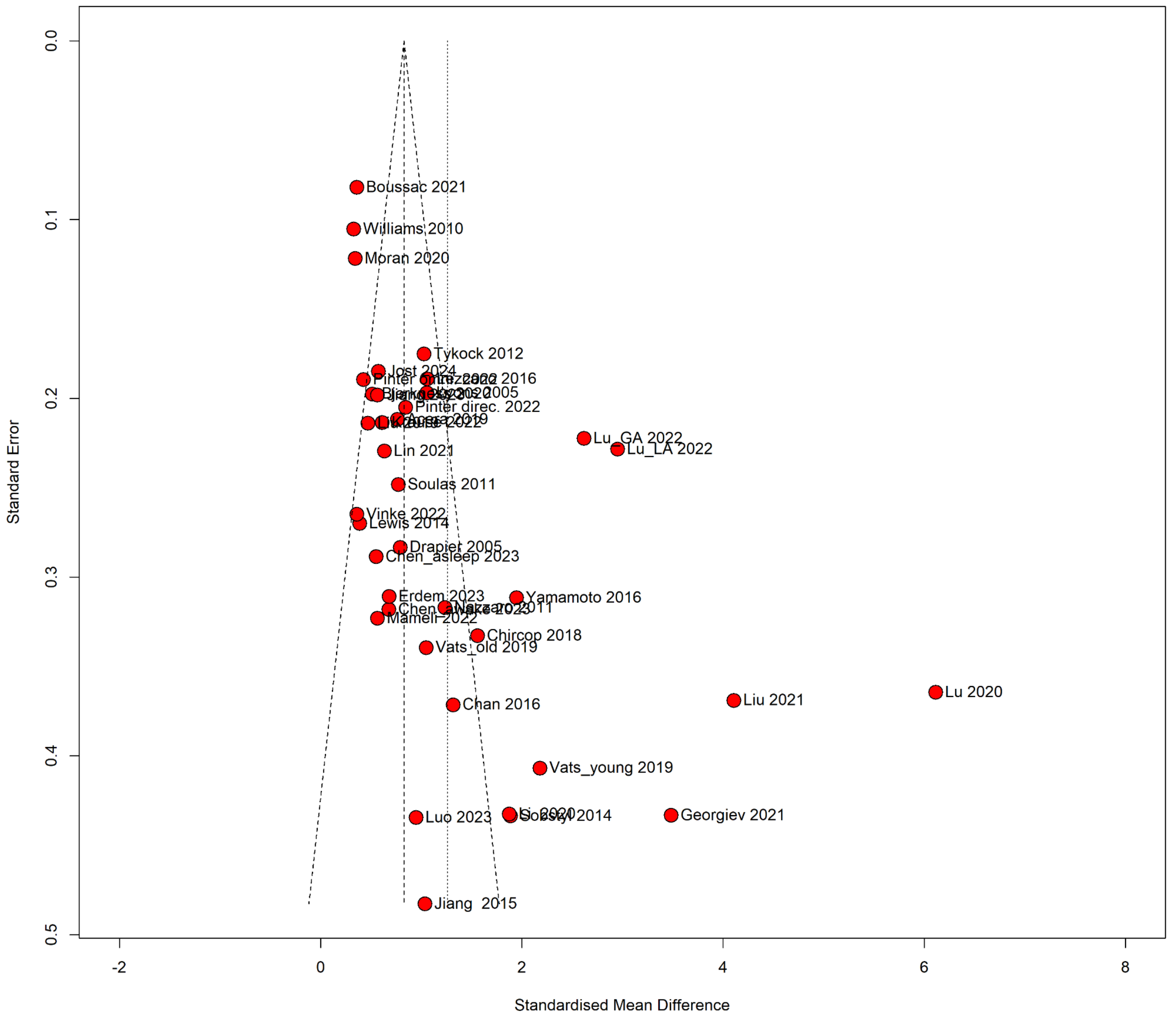

Supplement: Supplementary file 1 — Supplementary TABLE S1. Baseline characteristics of included studies. Supplementary Figure S1. Forest plot of the follow‐up period of 12 months showing the SMD and the weights for the random effects analysis of each study. DBS, deep brain stimulation; SMD, standardized mean difference. Supplementary Figure S2. Meta‐regression modeling for the impact of mean baseline PDQ score and QoL at 12 months follow‐up (P = 0.011). Supplementary Figure S3. Meta‐regression modeling for the impact of mean baseline PDQ score and QoL at 24 months follow‐up (P = 0.772). Supplementary Figure S4. Critical appraisal of individual studies according to Cochrane's tools for assessing risk of bias in non‐randomized studies (ROBINS‐I). Supplementary Figure S5. Critical appraisal of individual studies according to the Cochrane Collaboration's tool for assessing risk of bias in randomized trials (RoB 2). Supplementary Figure S6. Funnel plot of the follow‐up period of 12 months showing signs of asymmetry, indicating small‐study effect, confirmed by Egger's test (P = 0.0003). [file MDC3-12-577-s001.pdf]
